# Regulation of the renin-angiotensin-aldosterone system by cyclic nucleotides and phosphodiesterases

**DOI:** 10.3389/fendo.2023.1239492

**Published:** 2023-08-22

**Authors:** Stepan Gambaryan, Sanika Mohagaonkar, Viacheslav O. Nikolaev

**Affiliations:** ^1^ Sechenov Institute of Evolutionary Physiology and Biochemistry, Russian Academy of Sciences, Saint Petersburg, Russia; ^2^ Institute of Experimental Cardiovascular Research, University Medical Center Hamburg-Eppendorf, Hamburg, Germany; ^3^ German Center for Cardiovascular Research (DZHK), partner site Hamburg/Kiel/Lübeck, Hamburg, Germany

**Keywords:** cAMP, cGMP, phosphodiesterase, renin, aldosterone

## Abstract

The renin-angiotensin-aldosterone system (RAAS) is one of the key players in the regulation of blood volume and blood pressure. Dysfunction of this system is connected with cardiovascular and renal diseases. Regulation of RAAS is under the control of multiple intracellular mechanisms. Cyclic nucleotides and phosphodiesterases are the major regulators of this system since they control expression and activity of renin and aldosterone. In this review, we summarize known mechanisms by which cyclic nucleotides and phosphodiesterases regulate renin gene expression, secretion of renin granules from juxtaglomerular cells and aldosterone production from *zona glomerulosa* cells of adrenal gland. We also discuss several open questions which deserve future attention.

## 1 Introduction

**Figure 1:**
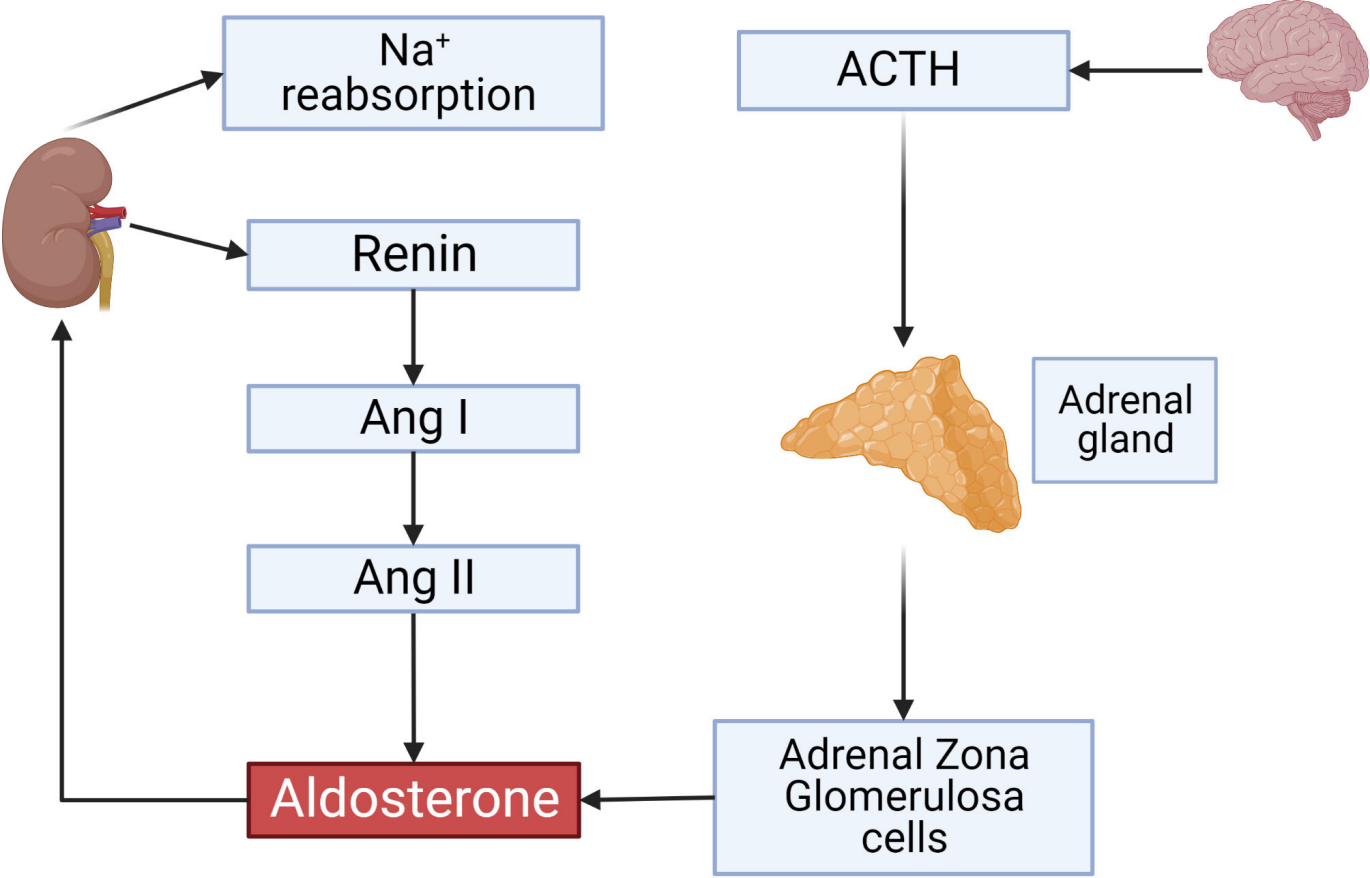
Overview on the renin-angiotensin-aldosterone System. ACTH secreted by the anterior pituitary gland stimulates aldosterone production from adrenal gland *zona glomerulosa* (ZG) cells. Renin secreted by JG cells of the kidney and its activity is a rate-limiting step controlling Angiotensin II concentrations which also activate aldosterone production. Aldosterone initiates sodium reabsorption from the kidney. This and all other figures were made using BioRender.

The renin-angiotensin-aldosterone system is one of the key players in the regulation of blood volume and blood pressure. Dysfunction of this system is connected with cardiovascular and renal diseases (1, 2). The main regulatory mechanism responsible for the activity of RAAS is a cascade of proteolytic enzymes that cleaves circulating angiotensinogen by renin to generate angiotensin I (Ang I), followed by subsequent cleavage of Ang I by angiotensin converting enzyme (ACE) to angiotensin II (Ang II). Angiotensinogen, the only precursor of all biologically active angiotensin peptides, is predominantly produced by the liver and its production is controlled by several hormones including estrogens, steroids, and thyroid hormones (**Figure 1**) (3). Historically, Ang II acting through Ang II receptors (AT-Rs), which belong to the G protein coupled receptor family (GPCR) was considered as a main regulator of RAAS system. This concept was revisited after discovery of angiotensin converting enzyme type 2 (ACE2) which generates Ang-(1-7). This short peptide acts at its specific GPCR called Mas receptor and counterbalances the vasoconstrictor role of Ang II (4–6).

Renin activity is a rate-limiting step controlling the activity of the RAAS. Juxtaglomerular (JG) cells located at afferent arterioles of the kidney glomeruli are the main source of renin secretion into the blood stream (7, 8). Generally, renin production from JG cells is controlled by renal perfusion pressure, the tubular sodium chloride concentration sensed by macula densa cells and negative feedback loops involving blood pressure, sodium balance and Ang II concentration (9). At the cellular level, cyclic nucleotides, 3’,5’-cyclic adenosine monophosphate (cAMP) and 3’,5’-cyclic guanosine monophosphate (cGMP) are strongly involved in the regulation of renin gene expression and release from JG cells.

Aldosterone is the next important player of RAAS which is synthesized and released by *zona glomerulosa* (ZG) cells of adrenal gland (**Figure 1**). Aldosterone, in addition to Ang II, is involved in the hemostatic regulation of blood pressure by controlling plasma sodium and potassium concentrations which are the main determinants of blood volume. Two classical pathways are involved in the regulation of aldosterone synthesis and release. Firstly, adrenocorticotropic hormone (ACTH) binds to the Gs-coupled melanocortin type 2 receptor (MC_2_R) and initiates cAMP synthesis. Secondly, Ang II and potassium concentrations in the plasma by different mechanisms can increase intracellular calcium (10–12).

## 2 Cyclic nucleotide signaling and phosphodiesterases

**Figure 2:**
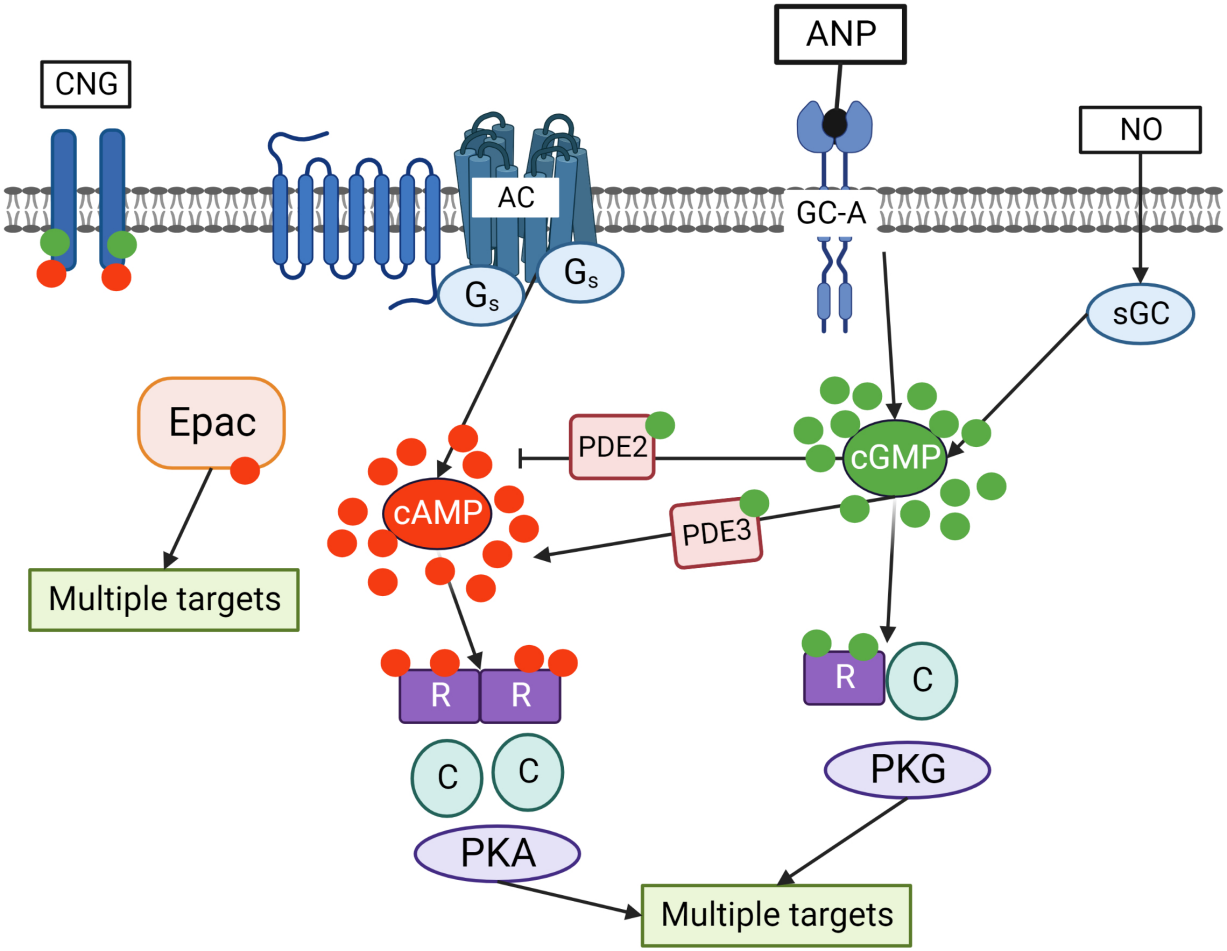
Crosstalk of cyclic nucleotide signaling pathways in cells. cGMP is synthesized by two independent pathways including transmembrane guanylate cyclases (GC-A) stimulated by ANP and sGC stimulated by NO. Adenylate cyclase (AC), which is activated by Gs coupled seven transmembrane receptors, is the main source of cAMP in the cells. cGMP by binding to PDE2 accelerates cAMP hydrolysis and by binding to PDE3 inhibits cAMP hydrolysis. Binding of cGMP to PKG and cAMP to PKA activates the kinases which phosphorylate multiple substrates regulating various intracellular processes. In addition, cAMP can directly bind to exchange protein activated by cAMP (Epac) that also has multiple targets in the cells. Cyclic nucleotides gated (CNG) channels are important mediators of cGMP/cAMP signaling in the cells.

In mammalian cells, cAMP is synthesized from ATP by adenylate cyclases (ACs) from 10 different families, regulated by G-protein coupled receptors (GPCRs), intracellular calcium and bicarbonate. cGMP is produced from GTP by two types of guanylate cyclase. The first one is the nitric oxide (NO) sensitive or the so-called soluble guanylate cyclase (sGC), which is directly activated by NO. The second one is a family of particulate guanylyl cyclases (pGCs) which are membrane receptors for natriuretic peptides (13–15). cAMP acts in cells primarily by activating cAMP dependent protein kinase (PKA), exchange protein directly activated by cAMP (Epac), or cyclic nucleotide gated (CNG) channels. cGMP can activate cGMP dependent proteins kinase (PKG) or CNG channels. PKA and PKG phosphorylate multiple substrates regulating various intracellular processes (**Figure 2**).

Cyclic nucleotides are degraded by PDEs which are hydrolyzing enzymes converting cAMP and cGMP to monophosphates. Over 100 PDE isoforms are generated by alternative splicing of more than 20 genes. PDEs have been classified into eleven families based on substrate specificity and regulatory properties (14–21). This high versatility leads to very precise regulation of cyclic nucleotide levels in various organ systems (14, 15). PDEs are also critically involved in the compartmentalization of cAMP and cGMP signaling in various subcellular nano- or microdomains which determine signal output by local substrate regulation (16). The response by each of the cyclic nucleotides in amplitude, space and time is controlled by their degradation via respective PDEs (17, 18). PDEs 4, 7 and 8 are highly specific for cAMP hydrolysis, whereas PDEs 5, 6 and 9 are cGMP specific. There are also several dual-specific PDEs such as PDE1, 2, 3, 10 and 11 (19–21).

Some dual-specific PDEs are also involved into the cross-talk between both cyclic nucleotides. For example, cGMP can activate PDE2 via an N-terminal regulatory domain, thereby promoting cAMP degradation and enabling a negative cGMP-to-cAMP cross-talk. Also, cGMP can bind to the catalytic domain of PDE3 with high affinity causing a negative cGMP-to-cAMP cross-talk and impaired cAMP degradation (22–26) (**Figure 2**).

Studies have reported that disruption in the expression of various PDEs or mutations in the PDE genes can lead to diseases including cancers, cardiovascular, neuronal, and other pathologies (18). Therefore, the role of PDEs and integration into the cAMP and cGMP pathways along with their crosstalk is essential to multiple physiological systems (27).

## 3 Regulation of RAAS by cyclic nucleotides and PDEs

### 3.1 Renin

**Figure 3:**
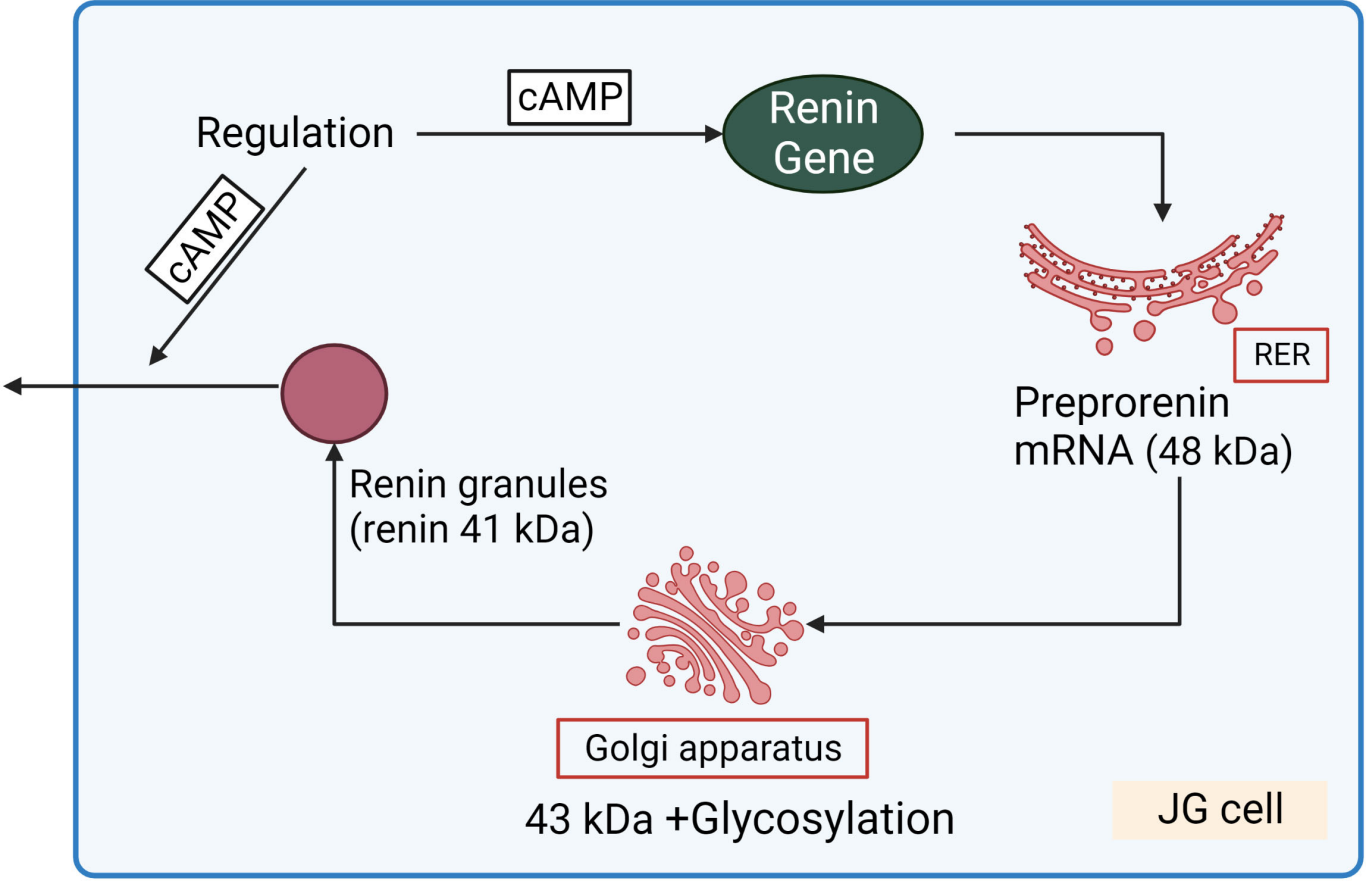
Renin synthesis and secretion in JG cells. Translation of renin mRNA at the rough endoplasmic reticulum (RER) gives rise a 48 kDa protein (preprorenin) from which the pre-(signal)-peptide is enzymatically cleaved in the Golgi-apparatus, and enzymatically inactive prorenin is then glycosylated and stored in the renin granules. Within the granules, prorenin (43 kDa) is proteolytically cleaved to yield enzymatically active renin (41 kDa). cAMP stimulated both renin gene expression and release of renin granules.

**Figure 4:**
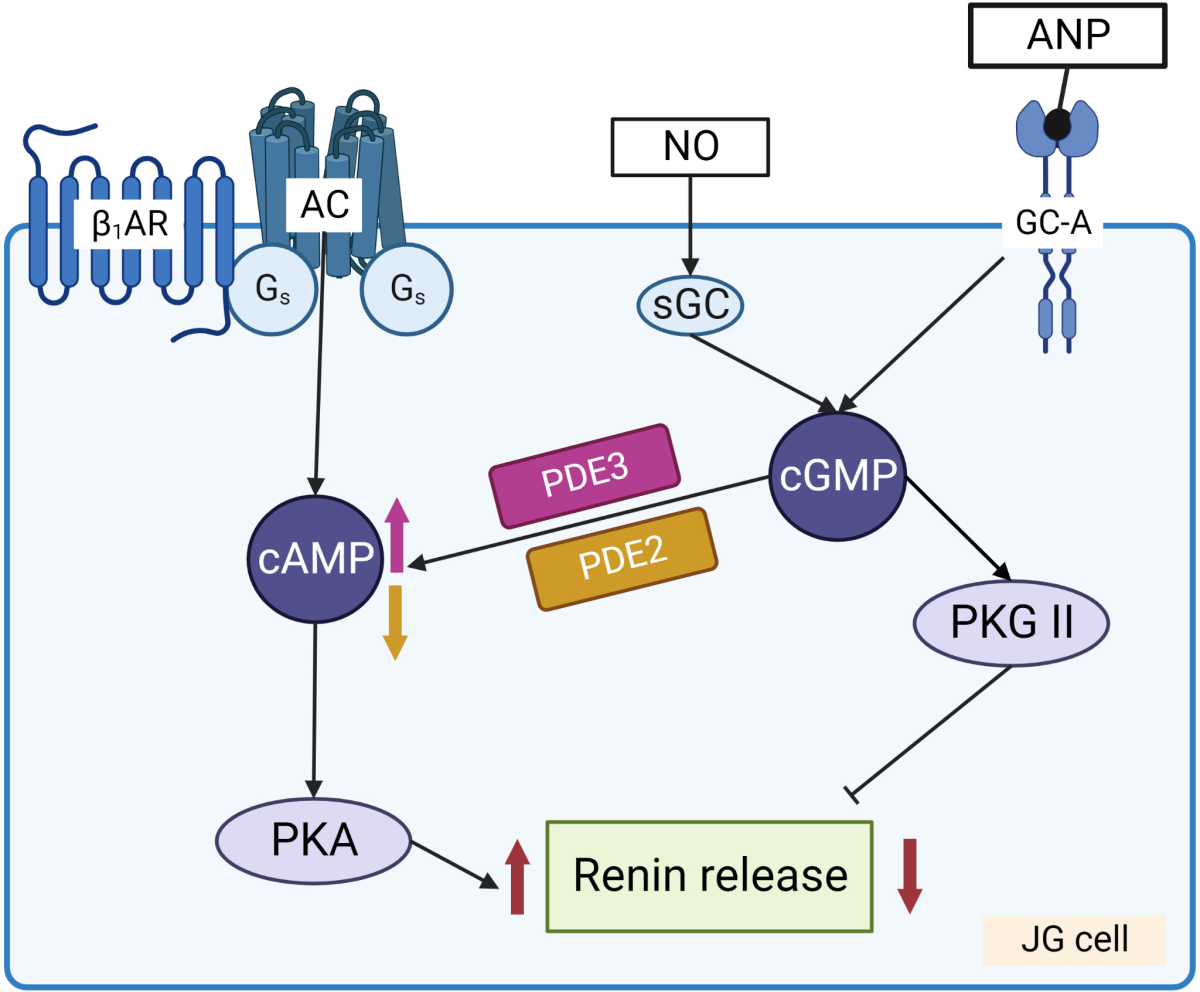
Regulation of renin release from JG cells by cyclic nucleotides. Stimulation of adenylate cyclase (AC) by Gs coupled receptors, such as β_1_-adrenergic receptor (β_1_-AR) and prostaglandin receptors, increases cAMP levels that activate PKA which enhances renin release from the granules. NO and ANP by stimulation of sGC and GC-A, respectively, increase cGMP which has dual effect on renin release. PKG II and PDE2 are involved in the inhibition of renin secretion, whereas cGMP mediated inhibition of PDE3 prevents cAMP degradation and stimulates renin secretion.

Renin concentration in plasma is regulated by the renin gene expression and release of renin from the renin containing granules in JG cells (**Figure 3**). These processes are regulated by second messengers including cyclic nucleotides and intracellular calcium. It is generally accepted that cAMP downstream of membrane receptors such as β_1_-adrenergic and prostaglandin receptors, is the main activatory signal for renin expression and release, whereas increase of calcium concentration inhibits it by so-called “calcium paradox” (28, 29). The effect of cGMP on JG cells is more complicated and still not fully understood. Several mediators of cGMP including PKG I, PKG II (30, 31) PDE2 and PDE3 (32, 33) are expressed in JG cells and cGMP could stimulate as well as inhibit renin gene expression and secretion. Atrial natriuretic peptide (ANP) cGMP-dependently inhibited renin release from JG cells (34), whereas inhibition of PDE3 by cGMP activates renin secretion (for details see below) (**Figure 4**).

### 3.2 Regulation of renin gene expression

Renin gene in human and mouse genome is localized in the chromosome 1. The 5’-flanking noncoding region plays a central role in regulating renin gene expression (35). Activation of the renin transcription is mainly mediated by the cAMP/PKA pathway (36, 37). In contrast, activation of protein kinase C and increase of intracellular calcium are involved in the suppression of renin expression (38, 39). cAMP-induced activation of the renin gene expression is initiated by PKA dependent phosphorylation of cAMP responsive transcription factors (CREB, CREM, and ATF1) that in phosphorylated form bind to CRE sequences in the proximal promoter and the kidney enhancer region (9). cAMP/PKA pathway is also critically involved in the control of basal transcriptional level of renin gene (40, 41). Deletion of Gs coupled receptors strongly decreased renin expression in mouse models (9, 37, 42). The question whether and how cGMP is involved in the regulation of the renin gene expression is still open. cGMP by inhibition of PDE3 enhances cAMP effect which in turn activates renin expression. Deletion of PKG II, but not PKG I, in mouse significantly enhanced renin mRNA levels (31). However, the molecular mechanisms of renin gene activation, whether it relates to the phosphorylation of the transcription factors by PKG II, or some other mechanisms are not clear. Nitric oxide (NO) by activation of soluble guanylate cyclase supports recruitment of renin producing cells to the glomerular arteriole (43), however the molecular mechanisms underlying these processes also not clear.

cGMP exerts dual effect on renin secretion (**Figure 4**). Activation of PKG II by 8-pCPT-cGMP, which is specific for PKG II and does not stimulate PDE3 (44) inhibited basal cAMP-stimulated renin secretion (30). Using PKG I and PKG II knock- out models (45) it was concluded that in mice, cGMP and PKG are involved in the acute regulation of renin release but not in the long-term regulation of renin gene expression and secretion (45). ANP increases cGMP in JG cells and inhibits renin release without affecting intracellular calcium concentration (34). Similarly, in several publications, inhibition of renin secretion by NO was demonstrated (46–48). On the other hand, some authors found a stimulatory role of NO on renin secretion (49–51) which is mediated by inhibition of PDE3 (7, 32, 52). The controversial results concerning effects of NO/sGC/cGMP pathway on renin secretion could be partly explained by different sources of NO that activate sGC in these cells. Increase of cGMP because of endothelial NO synthase (eNOS) activation by elevated renal perfusion and shear stress is probably involved in the inhibition of renin release by the activation of PKG II (31). Another source of NO for JG cells is the neuronal NO synthase (nNOS). nNOS is expressed in macula densa cells and upregulated by sodium restriction (52–55) that could be involved in activation of renin secretion by inhibition of PDE3 activity. Such controversies also exist in the remodeled microdomains of diseased hearts having altered cAMP compartmentalization (56). Since PDEs control the amplitude and the duration of cyclic nucleotide responses (17, 18), even small alterations in their activities can lead to dramatic remodeling (17, 27, 57–60). However, whether similar mechanisms are present in JG cells where cGMP downstream of particulate guanylate cyclase (activated e.g. by ANP) or NO derived from different NOS isoforms (eNOS, nNOS) remains to be elucidated.

### 3.3 Regulation of renin secretion by PDEs

From eleven PDE enzyme families, expression of only PDE1, PDE2, PDE3 and PDE4 was detected in JG cells (7). Although expression of PDE5 and PDE9 has not been directly demonstrated in JG cells, inhibition of PDE5 by sildenafil in two groups of normotensive humans with and without sodium intake restriction elevated renin secretion (61). Similarly, in patients with liver cirrhosis and ascites, sildenafil increased renin plasma levels (62). Inhibition of PDE5 by zaprinast in a rat model increased plasma renin activity six-fold without any effects on blood pressure and renal blood flow (33). On the other hand, in wild type and eNOS knock-out mice, inhibition of PDE5 by zaprinast did not change plasma renin concentration in both phenotypes (55). Inhibition of PDE9 by low concentrations of PDE9-I and PF-04749982 had no effect, and high concentrations of PDE9 inhibitors acutely increased plasma renin activity in sheep (63). However, all these data were obtained *in vivo* on human and animal models. Therefore, the question whether the effects of PDE5 and PDE9 inhibitors are connected with a direct action on JG cells or are mediated by systemic kidney or blood pressure responses is still open. In the literature, we could not find any direct indications that PDE5 and PDE9 are expressed in JG cells, and this question still needs further clarification.

Calcium-calmodulin-dependent PDE1 family, which includes PDE1A, PDE1B and PDE1C subfamilies, is expressed in different cells (21). The PDE1A and PDE1B have a higher affinity for cGMP than for cAMP, whereas PDE1C has an equal affinity for both cAMP and cGMP (64). PDE1C is the only subfamily expressed in JG cells. Elevated intracellular calcium by activation of PDE1C and inhibition of AC5 and AC6 leads to a decrease in cAMP and renin secretion (65).

Expression of PDE3A, PDE3B and PDE4 in JG cells was demonstrated at the mRNA level. Inhibition of PDE4 by rolipram significantly enhanced basal and forskolin induced renin secretion from isolated JG cells, suggesting an important role of this PDE family. PDE3A mRNA levels were higher than those of PDE3B and PDE4 in freshly isolated cells (7). Supporting this result, functional significance of PDE3, as a cGMP-inhibited PDE family, for the regulation of renin gene expression and secretion was demonstrated by numerous other reports reviewed in detail elsewhere (8).

In summary, cyclic nucleotides cAMP, and cGMP, along with their downstream protein kinases and several PDEs, play a crucial role in the regulation of renin gene expression and renin secretion from JG cells. However, there are many open questions especially concerning compartmentalization of cyclic nucleotide signaling in these cells and specific PKA/PKG substrates which are responsible for renin regulation.

## 4 Aldosterone

In mammals, aldosterone biosynthesis occurs mainly in the ZG cells of adrenal gland. Cholesterol is a common precursor for synthesis of all steroid hormones including aldosterone. Synthesis of the steroid hormones starts from mobilization of cholesterol esters from intracellular lipid droplets and their enzymatic hydrolysis to free cholesterol by cholesterol ester hydrolase (CEH). Aldosterone is synthesized by a series of enzymatic reactions that involve three cytochrome P450 enzymes and hydroxysteroid dehydrogenase. For initiation of the aldosterone synthesis, free cholesterol is transported to the outer mitochondrial membrane. Cholesterol then moves from the outer mitochondrial membrane, across the aqueous intra-membranous space, to the inner mitochondrial membrane where the side-chain cleavage enzymes system is localized. The movement across aqueous space is a rate-limiting step in aldosterone synthesis which is regulated by the expression and phosphorylation of steroidogenic acute regulatory protein (StAR) (66–69). In StAR knock-out mice, stimulation of the steroid hormone producing cells induced progressive accumulation of lipids within the steroidogenic cells and ultimately causing their death (70). Several mechanisms including cyclic nucleotides, intracellular calcium and potassium concentrations, activity of many protein kinases are involved in the regulation of aldosterone synthesis and production (12, 71). In this review, we focus mainly on the mechanisms of cyclic nucleotide and PDE dependent regulation of aldosterone synthesis.

### 4.1 Regulation of aldosterone by cyclic nucleotides

#### 4.1.1 cAMP pathways

**Figure 5:**
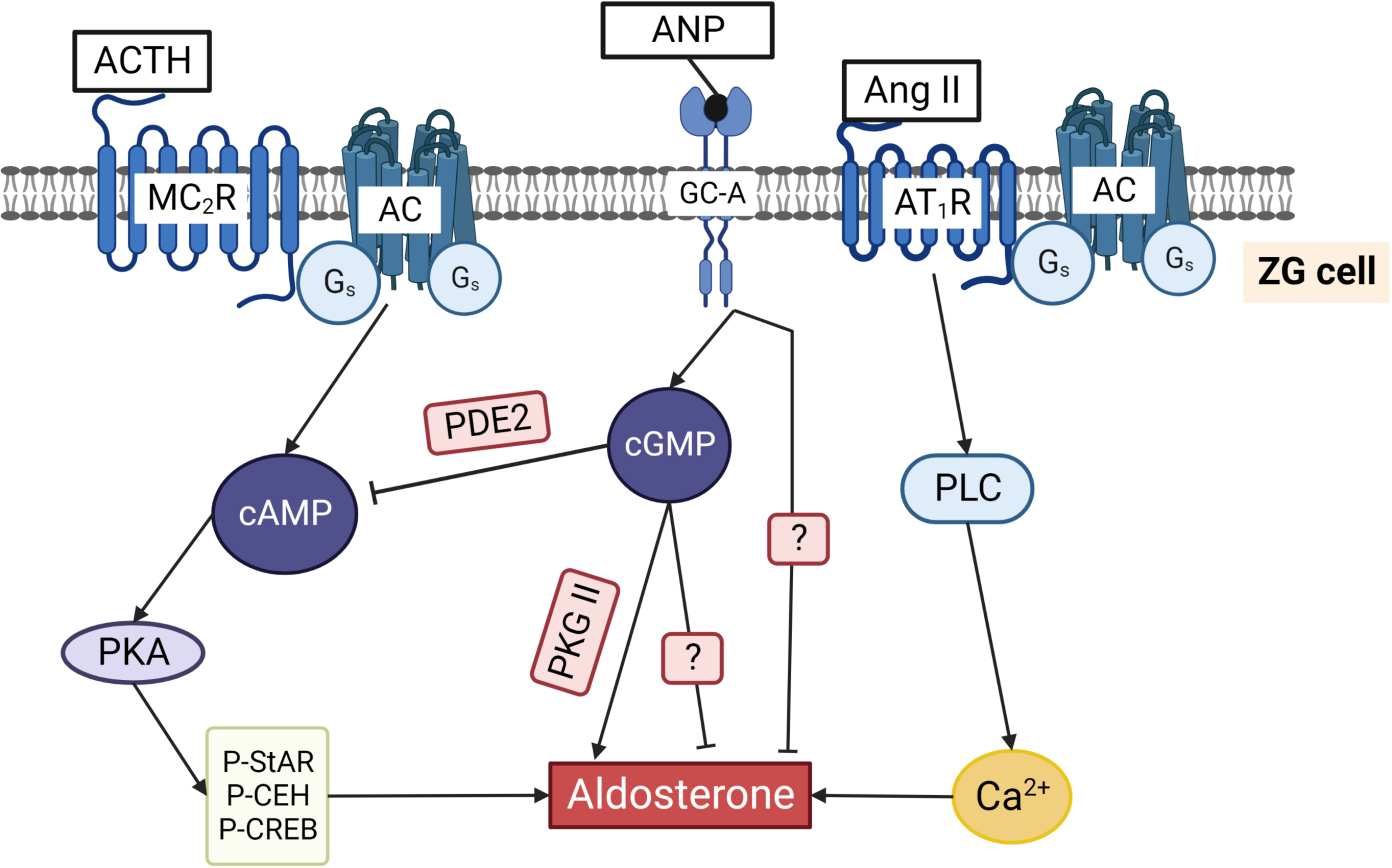
Regulation of aldosterone production in ZG cells by cyclic nucleotides. ACTH binding to the Gs coupled MC_2_R increases cAMP and activates PKA. Phosphorylation of cholesterol ester hydrolase (CEH), StAR, and several transcription factors stimulates aldosterone production. Ang II binds to G_q/11_ and G_i/o_ coupled AT_1_R that activates phospholipase C and increases cytosolic Ca^2+^ concentrations, thereby triggering aldosterone production. ANP by binding to GC-A increases cGMP that can inhibit aldosterone production by binding to PDE2 and reducing cAMP. Alternatively, cGMP can stimulate aldosterone production by activating PKG II. ANP inhibits not only ACTH/cAMP stimulated but also Ang II stimulated aldosterone production by a still unknown mechanism. The two question marks indicate that the exact pathways inhibiting aldosterone directly via cGMP and via ANP stimulated GC-A are still unknown.

ACTH by binding to its Gs-coupled MC_2_R increases cAMP concentration in ZG cells (**Figure 5**). Several AC families including the calcium activated AC-1 and AC-3, calcium inhibited AC-5/6, and the βγ- and PKC-sensitive AC-2/AC-4 are expressed in these cells (72). In addition to these AC families, soluble AC (sAC) is expressed in ZG cells and its activation enhanced cAMP in mitochondria and aldosterone production (73). Direct increase of the mitochondrial cAMP was demonstrated by mitochondria-targeted fluorescent biosensor, and knockdown or inhibition of sAC reduced mitochondrial cAMP and angiotensin II-induced aldosterone production (74). Activation of AC-1 and AC-3 by cAMP/PKA-mediated phosphorylation of L-type calcium channels is also involved in cAMP rise in ZG cells (75, 76). Ang II alone strongly stimulates aldosterone secretion via elevation of intracellular calcium (77, 78). This pathway can cross-talk to cAMP signaling in different ways including calcium activated and calcium inhibited ACs mentioned above. For example, elevation of intracellular calcium concentration by Ang II potentiated ACTH-mediated cAMP increase in ZG cells (79). However, in some reports, reduction of ACTH-induced cAMP production by Ang II (80–82) as well as no effect on cAMP (83) were also shown. In bovine ZG cells, stimulation by Ang II was not sufficient to increase cAMP and activate PKA (78). This discrepancy could relate to differences in intracellular mechanisms of cAMP synthesis in different species (human, bovine, rat ZG cells, human H295R adrenocortical cell line) and different experimental settings. cAMP mediated activation of PKA is important for aldosterone synthesis. PKA, by phosphorylation and activation of CEH, regulates the availability of free cholesterol, the initial substrate for aldosterone biosynthesis, which catalyzes the hydrolysis of stored cholesterol esters into free cholesterol and a fatty acid (84, 85). Phosphorylation of the transcription factor CREB by PKA activates transcription of enzymes responsible for aldosterone synthesis and StAR gene expression (**Figure 5**). Activation of StAR gene expression is under the control of multiple factors, including several protein kinases and transcription factors activated by PKA (86, 87). Transcription of StAR mRNA is also regulated by PKA. cAMP/PKA activation induces transcription of longer but less stable StAR mRNA, whereas under basal conditions, a shorter but more stable mRNA is transcribed (88, 89). Activity of StAR itself is stimulated by PKA phosphorylation. StAR protein contains two consensus PKA phosphorylation sites (S56/57, RRGS or RRSS, and S194/195 with RRGS motive) that are conserved among mammals (84). At least two PKA isoforms (PKA I and PKA II) are expressed in steroidogenic cells, and differential activation of these kinases by specific analogs revealed that activation of StAR gene expression is more dependent upon type I PKA, while the phosphorylation of StAR is mainly mediated by PKA II activation (90). From numerous A-kinase anchoring proteins (AKAPs) only two, namely AKAP121 and Optic Atrophy 1 (OPA1) were described in steroidogenic tissues. AKAP121 forms a complex with PKA R II subunit and StAR mRNA during the increase of intracellular cAMP levels, and this complex is important for translocation of StAR mRNA into mitochondria and for the prevention of StAR mRNA from degradation (90, 91). OPA1 in aldosterone producing cells is localized in mitochondria and in the cytosol. However, direct involvement of OPA1 in regulation of steroidogenesis has not been reported (92).

In addition to PKA, exchange proteins activated by cAMP (Epac1 and 2) and their effectors, small GTPases Rap1 and Rap2, are expressed in ZG cells (93). Epac mediated activation of Rap1 and 2 by ACTH, as well as direct Epac activation by specific cAMP analog 8-pCPT-2’-O-Me-cAMP had no effect on aldosterone production in bovine ZG cells (78). The lack of Epac effect on aldosterone production was confirmed in murine adrenocortical tumor cells (Y1) and in human H295R cell line. It was shown that the activation of Epac in these cells is involved in cytoskeleton integrity and cell migration but not aldosterone production (94). On the other hand, it was shown that Epac is downstream of mitochondrial sAC and mitochondrial cAMP, suggesting that Epac plays an essential role in the control of initial calcium uptake. It may also contribute to maintaining elevated mitochondrial calcium level. However, whether this function of Epac is directly related to the aldosterone production is not clear (73) and the question about possible direct effects of Epac activation on aldosterone production from ZG cells remains open.

Several isoforms of Calcium/Calmodulin-dependent Protein Kinases (CaMKs) are expressed in ZG cells and are strongly involved in the regulation of aldosterone production (95). By using *in vitro* kinase assays and experiments with intact bovine and rat ZG cells it was shown that cAMP directly stimulated CaMK activity which was independent of PKA and Epac/Rap1 signaling systems. These data suggests that in ZG cells, some other target(s) of cAMP might be involved in the regulation of CaMK activity (78).

#### 4.1.2 cGMP pathways

Synthesis of cGMP is mediated by activation of natriuretic peptide receptors and soluble NO-sensitive guanylate cyclase. ANP by the activation of its receptor (GC-A) significantly increases cGMP level in ZG cells, whereas the expression and activity of sGC in these cells was not directly shown. NO donors inhibited aldosterone production in bovine ZG and H295R cells without increasing intracellular cGMP, and the sGC inhibitor ODQ did not prevent this NO effect. Additional proof that cGMP is not involved in NO-mediated aldosterone production was based on the data that membrane permeable cGMP analogs had no effect on aldosterone production (96, 97). However, it should be mentioned that ZG cells are full of lipid granules and cGMP analogs are highly lipophilic, therefore higher concentrations of analogs have to be used in ZG cells. For example, much lower concentrations of cGMP analogs are already active in JG cells and platelets (30, 98) than in ZG cells (99). In several cell types, including vascular smooth muscle cells, endothelial cells and neurons, extensive cross-talk between Ang II and NO signaling has been described. Ang II, by several intracellular mechanisms including intracellular calcium can activate eNOS leading to increased NO production. On the other hand, elevated NO levels can downregulate AngII AT1-receptor mRNA and protein expression (100, 101). eNOS expression in sheep and rhesus ZG cells was demonstrated by immunohistochemistry (102), suggesting that this mechanism could potentially operate in adrenal cells and modulate aldosterone secretion. However, the question whether eNOS is expressed in ZG cells of other species including mice, rats and humans, and the relevance of this crosstalk between Ang II and NO is ZG cells deserve further investigation.

PKG I and PKG II are expressed in rat adrenal cortex. PKG I expression is restricted only to the capsule of the adrenal gland and blood vessels, whereas PKG II is expressed solely in ZG cells. Low sodium intake in rats induces hyperplasia and hypertrophy of ZG cells (103) and upregulates PKG II expression in these cells without changes in PKG I expression. This indicates that PKG II is important for functional activity of ZG cells. cGMP generated upon activation of GC-A is a well-known inhibitor of aldosterone production which is mediated by the stimulation of PDE2 (see below). To demonstrate the effects of PKG II on aldosterone production, selective PKG activator (8-pCPT-cGMP) and inhibitor (Rp-8-pCPT-cGMPS) in concentrations that do not affect PDEs (104) were used. Activation of PKG II stimulated basal as well as ACTH and Ang II-stimulated aldosterone production, and inhibition of PKG II significantly reduced it. These effects were not detected in H295R cells which do not express PKG II. Overexpression of PKG II by adenovirus in rat ZG cells strongly enhanced its effect on activation of aldosterone production. StAR protein was phosphorylated by PKG II *in vitro*. However, whether it could be phosphorylated *in vivo* is still not known. In contrast to the PKA effect, activation of PKG II did not induce StAR gene expression, and it was concluded that PKG II activity is important for maintaining basal level of aldosterone production in rats (99). PKG II is also expressed in mouse ZG cells, and the authors did not find similar function of this kinase in the regulation of aldosterone production by using PKG II knock-out mouse model. Basal plasma aldosterone levels were similar in wild-type and PKG II knock-out mice. The authors made an interesting observation that *in vivo* injection of ANP decreased ACTH-stimulated aldosterone secretion in wild-type mice and had no effect in PKG II knock-out mice, but the molecular mechanism of this effect is not clear (105). However, the results on mice and rats are not comparable because rat experiments were performed in isolated ZG cells (99), whereas mouse experiments were conducted *in vivo* (105), and other factors could influence the effects of PKG II on aldosterone production. PKG II is not expressed in H295R cells (see above), and there are no data whether this kinase is expressed in other aldosterone producing cells (bovine, human) or in other cell types used for analysis of intracellular mechanisms of regulation of aldosterone production. Therefore, the question whether this kinase is involved in regulation of aldosterone production in other species except mouse and rat remains open.

Shortly after discovery of ANP (106), several reports described its inhibitory effect of ANP on aldosterone production from ZG cells (**Figure 5**) (107–112). cGMP-independent inhibition of aldosterone production was initially postulated in all these reports based on the lack of inhibitory effect of cGMP analogs (8-Br-cGMP, Db-cGMP). Similarly, inhibition of StAR gene expression by ANP was not mimicked by cGMP analogs (113) (please see also above for the information regarding the problem for use of cGMP analogs in ZG cells). In 1991, high expression of PDE2 in bovine ZG cells was described. It was shown that the inhibitory effects of ANP on cAMP-stimulated aldosterone production is mediated by activation of PDE2 and strong decrease of cAMP concentration (114). After this, the predominant role of cGMP stimulated PDE2 as a mediator of ANP inhibitory effects in ZG cells was confirmed by several studies (see next chapter).

### 4.2 Regulation of aldosterone by PDEs

From all known PDE families, only PDE2 expression has been described in aldosterone producing cells. Expression of PDE2 is very high in adrenal ZG cells, and increase of cGMP can strongly inhibit aldosterone production (**Figure 5**) (114). For real time *in situ* monitoring of PDE2 activity in bovine ZG cells, a genetically encoded fluorescent cAMP biosensor was used. ANP-induced increase of cGMP activated PDE2 and induced a rapid decrease of intracellular cAMP within a few seconds. Moreover, the kinetics of ANP-stimulated PDE2 activity was much faster than the speed of ACTH-induced cAMP synthesis in these cells, revealing high catalytic activity and fast action of PDE2 in regulating cAMP levels in ZG cells (115). The same cAMP sensor was used in H295R cells to show the predominant role of PDE2 in the inhibition of aldosterone production (2). In humans, mutations of PDE2A and PDE3B variants were associated with familial primary aldosteronism and bilateral adrenal hyperplasia. However, high aldosterone concentrations were detected only in patients with PDE2A mutation, whereas patients with PDE3B mutation had abnormal cortisol levels (116). Cushing syndrome, adrenal tumors, and hyperplasia of adrenal glands in humans are also associated with the mutations in PDE8 and PDE11 genes (117–120). Different isoforms of PDE8 (121) and PDE11 (117, 120) are strongly expressed in adrenal *zona fasciculata* cells and we could not find in the literature whether any other PDEs apart from PDE2 are expressed and directly involved in the regulation of aldosterone production in ZG cells.

## 5 Conclusions

Regulation of RAAS is under the control of multiple intracellular mechanisms. Cyclic nucleotides and PDEs serve as the major regulators of this system by controlling expression/activity of renin and aldosterone production. In general, it is accepted that cAMP/PKA pathway is a positive regulator of RAAS, whereas cGMP is mainly regarded as a negative regulator, inhibitory pathway. However, there are still many open questions which deserve further investigation. In JG cells, phosphorylation of transcription factors by PKA activates renin gene expression but the substrates involved in renin granule release are mostly unknown. Regulation of gene expression by PKG II was described for other cell types (37, 122), however, whether PKG II is also involved in regulation of renin gene expression is not known. In JG cells, PKG II is localized in renin containing granules and its activation inhibits renin secretion (30). However, it is still unknown which substrates are phosphorylated by PKG II in these cells. Discrete subcellular microdomains confine cyclic nucleotide signaling which is highly compartmentalized. This gives freedom to the PDEs to control local kinase activities such as phosphorylation of specific PKA and PKG substrates. Not only does this limit the activity but also allows the cross talk between the signaling pathways, which is well understood in cardiomyocytes but still under investigation in many other cell types (57). Compartmentalization of cyclic nucleotides is an important regulator of their function in other cell types, but there is no information about compartmentalization of cAMP/cGMP in JG cells. Also, information concerning Epac and AKAPs in these cells is very scarce. Similar questions could be addressed in aldosterone producing cells. In addition, very little is known concerning the expression and function of different PDEs (except PDE2) in ZG cells. Whether other PDEs are expressed and involved in the regulation of aldosterone production is still not known.

## Author contributions

All authors listed have made a substantial, direct, and intellectual contribution to the work and approved it for publication.
